# Edge percolation centrality: A new measure to quantify the influence of edges during percolation in networks

**DOI:** 10.1371/journal.pone.0331475

**Published:** 2025-09-26

**Authors:** Christina Durón, Hannah Kravitz, Moysey Brio

**Affiliations:** 1 Natural Science Division, Pepperdine University, Malibu, California, United States of America; 2 Fariborz Maseeh Department of Mathematics and Statistics, Portland State University, Portland, Oregon, United States of America; 3 Department of Mathematics, University of Arizona, Tucson, Arizona, United States of America; Rutgers The State University of New Jersey, UNITED STATES OF AMERICA

## Abstract

Numerous centrality measures exist to quantify the influence of edges within a network, with edge betweenness being one of the more well-known measures. However, such measures are inadequate in network percolation scenarios (e.g., the transmission of a disease over a transportation network of highways) as they fail to consider the changing percolation states of edges over time. This paper addresses this limitation by extending percolation centrality, a measure originally developed to evaluate the influence of vertices during a percolation process (i.e., a dynamic spread of a contagion) in the network, to the edge level. The proposed measure, edge percolation centrality, captures both the topological connectivity of the network as well as the percolation states of the edges. Although the algorithm’s observed complexity of $O(|V{|}^{3.57})$ makes it computationally intensive, the utility of the proposed edge measure is evident in its application to both synthetic and real-world networks undergoing percolation processes.

## Introduction

In this work, ‘percolation’ refers to the dynamic spread of a contagion through a network — as in models of epidemiological transmission or information diffusion — rather than the removal of vertices or edges, as is common in studies of network robustness. The percolation of a contagion occurs in many real-world networks, including the diffusion of infectious diseases through contact networks [1,2], the transmission of pathogens between cities through transportation networks [3,4], and the spread of computer viruses across technological networks [5,6]. In each case, the contagion spreads along the edges of the network, triggering a percolation process that changes the states of the edges (and indirectly, the vertices they connect) over time. These edge states may be binary (e.g., active or inactive), discrete (e.g., susceptible or infected or recovered), or continuous (e.g., an infection density).

To mitigate contagion spread through the network, it is crucial to identify edges that contribute most significantly to its propagation (e.g., [7] studied contagion blocking by identifying edges to remove from social networks). However, limited resources for intervention (e.g., closing roads, monitoring data connections) demand strategic decisions, and edges (e.g., highways, airline routes) that are most vulnerable individually may not be those most influential to the spread. Therefore, effective intervention requires identifying edges that are influential in both their topological position within the network and their current percolation state. Since infected (percolated) edges exert a greater influence on the spread of the contagion than uninfected (not percolated) ones, an effective measure of the influence of an edge during this process must account for both its topological location and percolation state (e.g., [8] introduced an edge ranking algorithm to identify critical edges both in network connectivity and spreading dynamic). Unfortunately, standard edge centrality measures (e.g., a well-known measure is edge betweenness [9]) quantify influence based upon the static topology of the network and thus fail to capture the change in percolation states of the edges over time.

To address the limitations of static edge centrality measures in these contexts, the edge percolation centrality – an edge-centric analogue of the vertex percolation centrality developed by [10] – is proposed to quantify the influence of an edge to the overall percolation process in the network. Similar to its vertex counterpart, the edge percolation centrality incorporates both the topological position of an edge and its changing percolation state over time. Moreover, it integrates the edge betweenness centrality by explicitly accounting for percolated edges on relevant shortest paths. In fact, when all edges are fully percolated (or partially percolated to the same extent), then the edge percolation centrality is shown to be equivalent to the edge betweenness centrality. More broadly, the edge percolation centrality may be useful in scenarios where edge functionality is critical (e.g., identifying transportation pathways essential for disease propagation [8] or transmission lines whose failure could disrupt power grids [11]).

The structure of the paper is as follows. The ‘Relevant Centrality Measures and Definitions’ Section reviews relevant centrality measures and provides formal definitions. The ‘Edge Percolation Centrality’ Section introduces the edge percolation centrality measure. The ‘Computational Complexity’ Section analyzes the computational complexity of the proposed edge measure while the ‘Synthetic Example’ Section demonstrates its calculation on an artificial network. The ‘Results’ Section presents the results of simulations of contagions (one simple and one complex) on real-world networks while also illustrating how the measure could be used as a tool to mitigate contagion spread. Finally, conclusions and possible directions for future work are detailed in the ‘Discussion’ Section.

## Relevant centrality measures and definitions

Centrality measures are used to identify the most influential vertices (edges) within the network. Specifically, a centrality measure is a function which assigns a numerical value to each vertex (edge) within the network where a vertex (edge) with a higher centrality value is usually considered more influential than the other vertices (edges). Although many centrality measures have been proposed to rank vertices (edges) within a network according to their level of influence, one of the most well-known vertex-centric measures is the betweenness centrality (BC) [12]. By definition, the (normalized) betweenness of a vertex v∈V within a network G=(V,E) is the sum of the fraction of all-pairs shortest paths that pass through *v*,


BC(v)=1(|V|−1)(|V|−2)∑a,b∈Va≠v≠b|V|σa,b(v)σa,b

where $\sigma_{a,b}$ is the total number of shortest paths from vertex *a* to vertex *b* while $\sigma_{a,b}(v)$ is the total number of those paths that pass through *v*. Note *a* is the source vertex while *b* is the target vertex on the path. Intuitively, the betweenness centrality quantifies how much information is likely to flow through vertex *v*.

The (normalized) edge betweenness centrality (EBC) [9] of an edge e∈E, the analogue of betweenness, is similarly defined as


EBC(e)=1|V|(|V|−1)∑a,b∈Va≠b|V|σa,b(e)σa,b

where $\sigma_{a,b}(e)$ is the total number of shortest paths from vertex *a* to vertex *b* that pass through edge *e*. Intuitively, the edge betweenness centrality quantifies how much information is likely to flow through edge *e*.

Piraveenan et al. [10] define the percolation centrality for a given vertex, at a given time, as the proportion of percolated paths that go through that vertex. A ‘percolated path’, by their definition, is a shortest path between a pair of vertices where the source vertex *a* is percolated (e.g., infected). Formally, the percolation centrality (PC) of a vertex *v* at time *t* is


PCt(v)=1|V|−2∑a,b∈Va≠v≠b|V|σa,b(v)σa,b·xat[∑i∈V|V|xit]−xvt

where $x_{a}^{t}$ and $x_{v}^{t}$ denote the percolation states of the source vertex *a* and vertex *v* at time *t*, respectively.

## Edge percolation centrality

The edge percolation centrality of an edge *e*, at a given time *t*, is defined as the proportion of percolated paths that go through that edge. In this context, a ‘percolated path’ is a shortest path between a pair of vertices such that the source edge (first edge) is percolated and the path includes edge *e*. Formally, the edge percolation centrality (EPC) of an edge *e* at time *t* is


EPCt(e)=|E|−1|V|(|V|−1)∑a,b∈Va≠b|V|σa,b(e)σa,b·xst[∑i∈E|E|xit]−xet

where $x_{s}^{t}$ denotes the percolation state of the first edge (source edge) on a given shortest path from vertex *a* to vertex *b* at time *t*, and $x_{e}^{t}$ denotes the percolation state of edge *e* at time *t*.

In this context, an edge *i* in a non-percolated state (e.g., susceptible) at time *t* is denoted by $x_{i}^{t}=0$ while one in a fully percolated state (e.g., infected) at time *t* is $x_{i}^{t}=1$; an edge *i* in a partially percolated state at time *t* is $0<x_{i}^{t}<1$ (e.g., an infection density along a road).

If all edges are fully percolated (or partially percolated to the same extent) and $x_{s}^{t}=K\leq 1$ for all possible source edges as well as the edge *e* itself, then $x_{s}^{t}/\left(\left[\sum_{i\in E}^{\left|E\right|}x_{i}^{t}\right]-x_{e}^{t}\right)=1/(|E|-1)$ is constant in time such that


EPCt(e)=1|V|(|V|−1)∑a,b∈Va≠b|V|σa,b(e)σa,b=EBC(e)

In this case, all shortest paths become percolated paths since all edges are potential sources of percolation. This is a direct analogue of the result obtained by [10] where they showed that PC reduces to BC when all vertices are fully percolated (or partially percolated to the same extent).

### Edge hop distance

Let the edge hop distance (EHD) of an edge *e* with respect to edge state vector *x* be defined as the minimum shortest path distance from either endpoint of edge *e* to any vertex incident to an edge with a nonzero value in *x*. Then EHD measures the minimum number of steps required to reach an infected edge from *e*. If edge *e* itself has a nonzero state, its edge hop distance is defined to be zero. If no edges in the network are infected, EHD is set to infinity. In general, edges positioned closer to infected edges will have lower EHD values. In the final simulation detailed in the ‘Results’ Section, edge hop distance is included as a simple contrasting measure to edge percolation centrality. This measure complements EPC by highlighting different facets of edge significance – for example, sustained influence on transmission (EPC) versus dynamic geographic exposure (EHD).

## Computational complexity

To derive the computational demands of the edge percolation centrality algorithm, its time complexity is analyzed under both worst-case and best-case scenarios.

In the worst-case scenario, the network G=(V,E) is dense or highly redundant such that many vertex pairs are connected by an exponential number of shortest paths. For each of the |E| edges, the algorithm iterates over all |V|(|V|−1) ordered vertex pairs *a*,*b*. For each pair, all shortest paths are computed, which can yield up to $O(2^{\left|V\right|})$ distinct paths in the worst case. Each path requires checking whether edge *e* appears on it, which takes O(|V|) time, and identifying the source edge, which takes O(|E|) time. This leads to a total worst-case time complexity of


$$O(|E|\cdot |V|(|V|-1)\cdot 2^{\left|V\right|}\cdot (|V|+|E|))$$

which is super-exponential and becomes computationally infeasible even for moderately sized networks.

In the best-case scenario, the algorithm runs on a sparse network where every vertex pair is connected by exactly one shortest path. For each of the |E| edges, the algorithm iterates over all |V|(|V|−1) ordered vertex pairs *a*,*b*. Since there is only one shortest path per pair and the path length is at most O(|V|), the inner loop remains relatively efficient. The edge inclusion check and source edge identification steps contribute O(|V|) and O(|E|) time per path, respectively. Thus, the best-case time complexity is


$$O(|E|\cdot |V|(|V|-1)\cdot (|V|+|E|))\approx O(|E|\cdot |V{|}^{3}+|E{|}^{2}\cdot |V{|}^{2})$$

In sparse networks where |E|=O(|V|), this simplifies to $O(|V{|}^{4})$ which is still polynomial, but much more tractable than the worst case.

**Fig 1:**
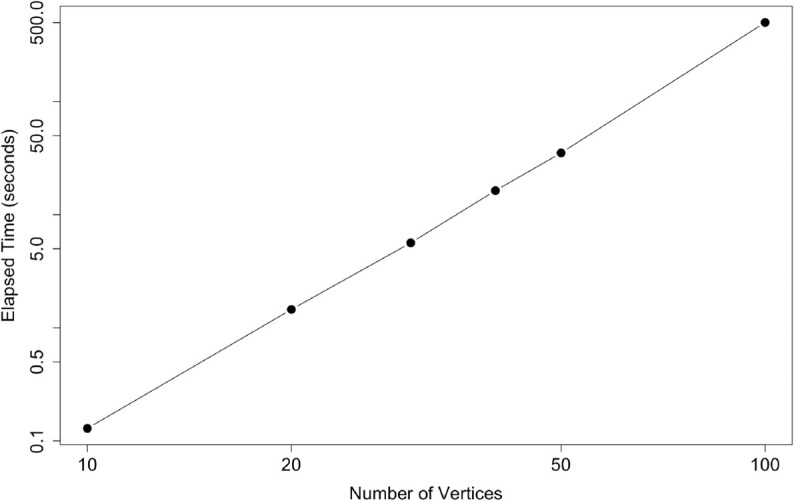
Elapsed time (in seconds) versus number of vertices |V| for the edge percolation centrality algorithm on scale-free networks with liner preferential attachment.

To empirically verify the computational complexity of the edge percolation centrality algorithm, a series of experiments were run on scale-free networks (i.e., networks characterized by the presence of vertices with a disproportionately large number of edges [13]) of varying sizes with linear preferential attachment. The network sizes ranged from |V|=10 to |V|=100 vertices, and for each size, the algorithm computed the edge percolation centrality given randomly assigned edge percolation state values in [0,1]. The computation time for each network was recorded using the system.time() function in R [14]. As seen in Fig 1, the observed complexity of the algorithm scales as $O(|V{|}^{3.57})$. The observed performance time is more efficient than the predicted best-case theoretical complexity, likely due to optimized underlying search algorithms in R. Although the algorithm’s runtime grows significantly as the network size increases, further optimization may improve the applicability of the measure on large networks.

## Synthetic example

**Fig 2:**
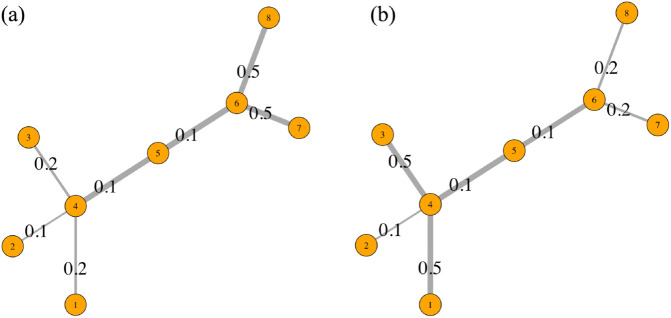
A synthetic network with |V|=8. Note that in (a), the edges in the right side of the network ($e_{6}=(v_{6},v_{7})$ and $e_{7}=(v_{6},v_{8})$) have high percolation states, whereas in (b), the edges in the left side of the network ($e_{1}=(v_{1},v_{4})$ and $e_{3}=(v_{3},v_{4})$) have high percolation states. The edge widths correspond to their edge percolation centrality values.

To illustrate the calculation of the edge percolation measure, consider the simple unweighted and undirected network in Fig 2 with percolation states ranging from 0.1 to 0.5 for each edge, as motivated by the synthetic network introduced in [10]. In Fig 2(a), the edges in the right side of the network (i.e., edges $e_{6}=(v_{6},v_{7})$ and $e_{7}=(v_{6},v_{8})$) have high percolation states, whereas in Fig 2(b), the edges in the left side of the network (i.e., edges $e_{1}=(v_{1},v_{4})$ and $e_{3}=(v_{3},v_{4})$) have high percolation states.

Consider edges $e_{4}=(v_{4},v_{5})$ and $e_{5}=(v_{5},v_{6})$. Both of these edges are centrally located in terms of network “traffic” and yield high edge betweenness values (16 and 15, respectively). Applying the edge percolation centrality to Fig 2(a), then edge *e*_4_ has the edge percolation centrality of $\text{EPC}(e_{4})=0.482$ while edge *e*_5_ has the edge percolation centrality of $\text{EPC}(e_{5})=0.509$. Although their edge percolation centrality is influenced by their topological placement, edge *e*_5_ has a slightly higher edge percolation centrality by being closer to the edges with higher percolation state values (i.e., edges *e*_6_ and *e*_7_). Applying the edge percolation centrality to Fig 2(b), then edge *e*_4_ has the edge percolation centrality of $\text{EPC}(e_{4})=0.482$ while edge *e*_5_ has the edge percolation centrality of $\text{EPC}(e_{5})=0.429$. While the topology remains unchanged, edge *e*_4_ now has a higher EPC value due to its proximity to edges with elevated percolation states (i.e., edges *e*_1_ and *e*_3_).

Consequently, the edge percolation centrality on a network with a fixed topology can vary significantly based on the percolated states of edges in the network.

## Results

### Simulation of simple contagion on a small real-world contact network

**Fig 3:**
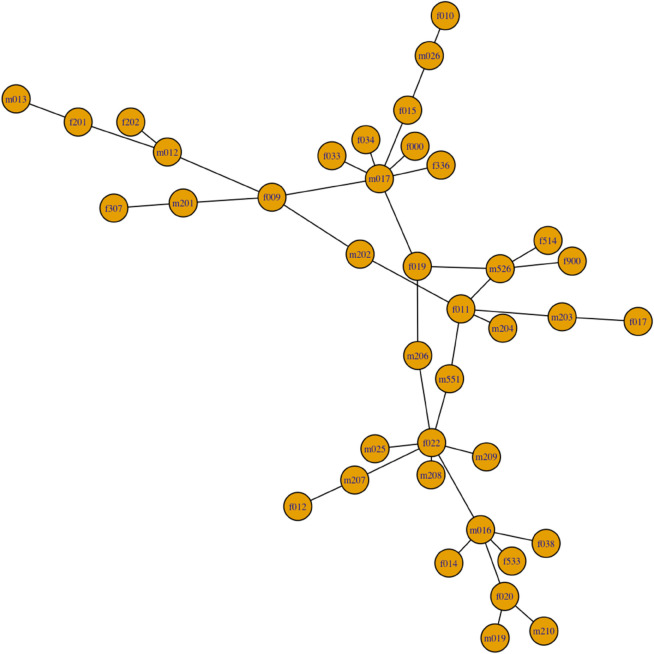
A real-world network with |V|=39 vertices and |E|=40 edges taken from the gonorrhea outbreak study in Alberta, Canada.

As motivated by the example introduced in [10], consider the small real-world network with |V|=39 vertices and |E|=40 edges (see Fig 3) which depicts the largest component of the gonorrhea outbreak study in Alberta, Canada [15]. To evaluate the edge percolation centrality as a generic centrality measure for contagion spread, a simple contagion model is simulated over *t* = 40 time steps, with the contagion initiating from a specific peripheral edge.

**Fig 4:**
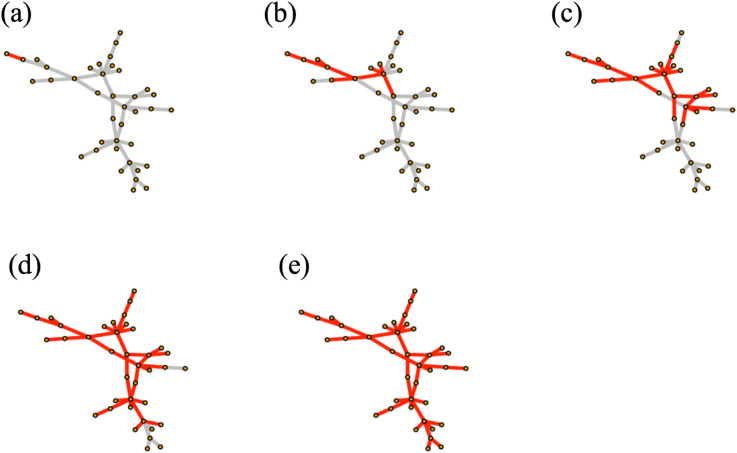
The contagion spreading on the Alberta model network at time step (a) $\text{t}={\text{t}}_{initial}=1$, (b) t=10, (c) t=20, (d) t=30 and (e) $\text{t}={\text{t}}_{final}=40$. Red edges are fully percolated (infected) while gray edges are not percolated (susceptible).

For example, consider a specific simulation in which a peripheral edge, $e_{1}=(\text{m013},\text{f201})$, (located in the top left hand side of the network) is the first edge to be fully percolated, $x^{1}(e_{1})=1$, and all other edges are not percolated, $x^{1}(e_{i\neq 1})=0$, at time step *t* = 1. At each time step *t*, edges become percolated with a transmission probability of *p* = 0.20, conditional on being adjacent to at least one percolated edge. As the contagion spreads, the percolation states of the edges are updated to *x*^*t*^(*e*) = 1 at time step *t*. Fig 4 illustrates the spread of the contagion through the network over time, beginning at *t* = 1 with the initial percolation of a single edge and culminating at *t* = 40 with the contagion fully propagated throughout the network.

**Fig 5:**
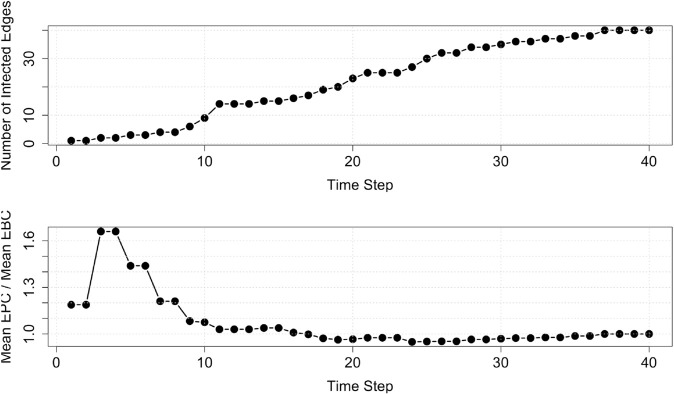
The number of infected edges (top) and the ratio of the average EPC and average EBC values, over time, for the Alberta model network.

The number of infected edges at each time step is shown in Fig 5 (top). The early peak in the infection curve occurs because the contagion initially spreads along peripheral edges before reaching edges that bridge densely connected parts of the network. Once these bridging edges become percolated, the infection quickly propagates to multiple neighboring edges, producing the rapid increase observed between *t* = 1 and *t* = 10.

To demonstrate how edge percolation centrality (EPC) differs from edge betweenness centrality (EBC) at each time step, consider the ratio of the averages between the measures for every edge. The ratio of averages over all |E| edges,


$$\frac{\frac{1}{\left|E\right|}\sum_{e\in E}\text{EPC}(e)}{\frac{1}{\left|E\right|}\sum_{e\in E}\text{EBC}(e)}=\frac{\sum_{e\in E}\text{EPC}(e)}{\sum_{e\in E}\text{EBC}(e)}$$

is also shown in Fig 5 (bottom).

At the beginning, *t* = 1, the EPC values, on average, do not significantly differ from EBC. At time *t* = 11, the contagion continues to spread across the network and the EPC values begin to converge to the EBC values. At time *t* = 37, every edge is percolated. At this time, the ratio $\sum_{e\in E}\text{EPC}(e)/\sum_{e\in E}\text{EBC}(e)$ approaches 1 such that EPC reduces to EBC for each edge (as derived in the ‘Edge Percolation Centrality’ Section).

**Fig 6:**
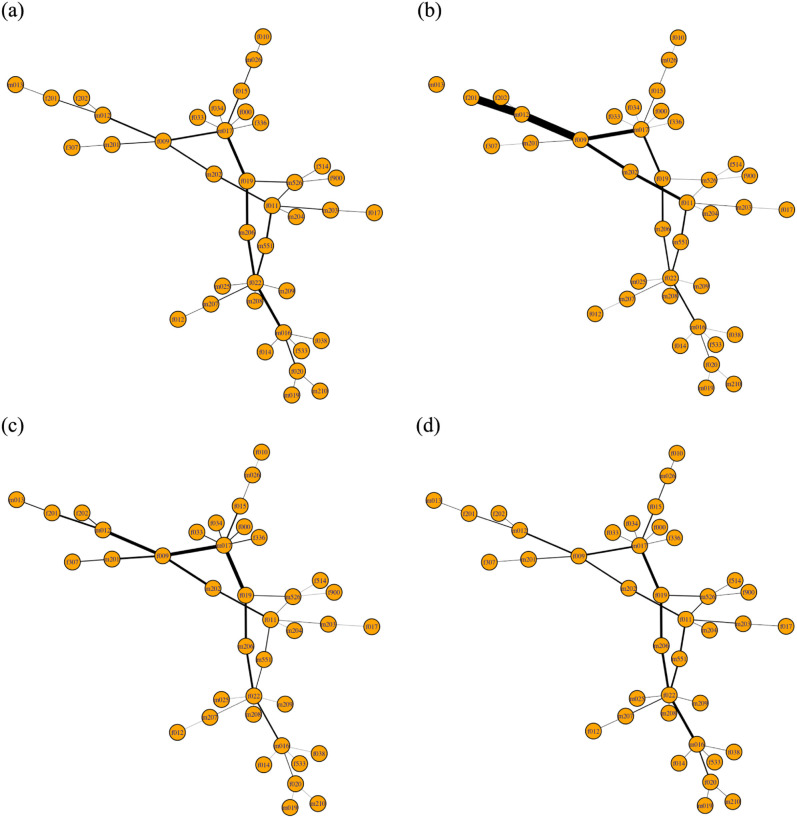
The edge betweenness and edge percolation centralities of the Alberta model network with |V|=39, with edge widths denoting the centrality values. The edge betweenness centrality (a) of the network is independent of time. The edge percolation centrality of the network at time (b) *t* = 1, (c) *t* = 11 and (d) *t* = 37.

The spread of the contagion is further illustrated in Fig 6, where the thickness of the edges reflects their respective centrality values. At *t* = 1 (see Fig 6(b)), edge $e_{2}=(\text{m012},\text{f201})$ exhibits the highest EPC, as it lies along the likely path of contagion spread and is therefore critical to its progression. The edge with the next highest EPC is $e_{4}=(\text{m012},\text{f009})$, due to its proximity to the initially percolated edge. In contrast, edge $e_{33}=(\text{m016},\text{f022})$, while centrally located, has a lower EPC because it is distant from the initial percolation source.

By *t* = 11 (see Fig 6(c)), the contagion has spread to fully percolate 14 edges. At this point, edge $e_{23}=(\text{m017},\text{f019})$ has the highest EPC due to its topological positioning and the percolation states of its neighboring edges.

At *t* = 37 (see Fig 6(d)), the contagion has fully spread across the network, with every edge in a percolated state. Here, edges *e*_23_ and *e*_33_ show the highest EPC values. Notably, when all edges are fully percolated, each edge’s EPC becomes equivalent to its EBC (see Fig 6(d) and Fig 6(a), respectively), as their importance is then determined purely by the network topology, just as with edge betweenness centrality. At this stage, edge *e*_2_ exhibits a reduced EPC, as it now resides on the periphery and no longer holds a unique position relative to the spread.

**Fig 7:**
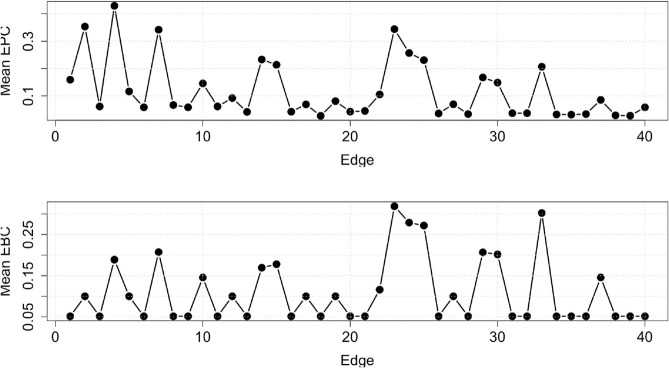
The average EPC (top) and average EBC (bottom), over time t=40, values of each edge in the Alberta model network.

Fig 7 shows the average EPC and average EBC values for each edge in the Alberta model network, computed over *t* = 40. While edges $e_{4},e_{2}$ and *e*_23_ exhibit the highest average EPC values, the highest average EBC values are observed for edges $e_{23},e_{33}$ and *e*_24_. Despite these differences, the two measures are strongly correlated, with a Spearman rank correlation of 0.92 between their rankings.

### Simulation of complex contagion spread on a small real-world road network

**Fig 8:**
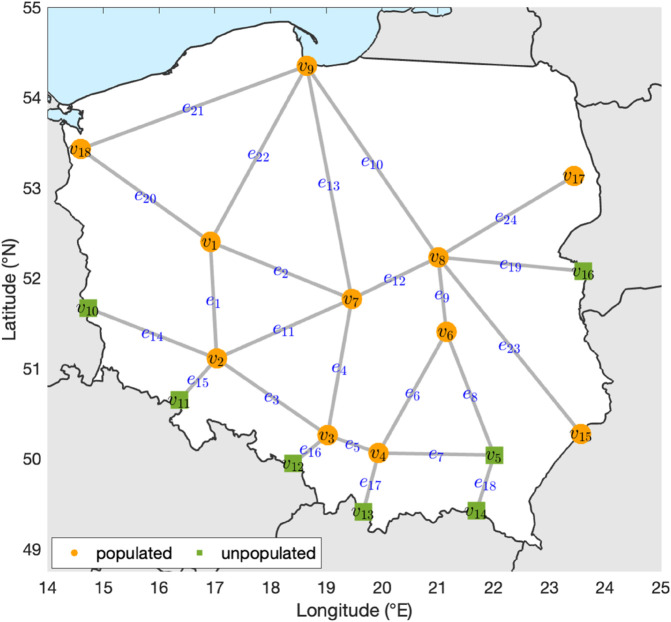
An approximation for the road network in Poland. Populated vertices are indicated by orange circles, and unpopulated ones by green squares. Country boundaries and coastlines are derived from public domain data provided by Natural Earth (http://www.naturalearthdata.com/). The network vertices and edges were generated by the authors.

As motivated by the example introduced in [16], consider a small real-world network with |V|=18 vertices and |E|=24 edges that approximates the major roads in Poland (see Fig 8). Table 1 provides the mapping of each vertex to its corresponding city or border crossing. Formally, this network is a metric graph – a type of flow network in which a metric is defined on each edge [17]. Consequently, the edge lengths (i.e., the metric) of the road network, shown in Table 2, represent the travel distances in kilometers between cities, as measured using Google Maps.

**Table 1:** The list of Polish cities and border crossings corresponding to each vertex in the road network.

Vertex	City	Vertex	City
$v_{1}$	Poznań	$v_{10}$	Germany border cross
$v_{2}$	Wrocław	$v_{11}$	Czechia border cross
$v_{3}$	Katowice	$v_{12}$	Czechia border cross
$v_{4}$	Kraków	$v_{13}$	Slovakia border cross
$v_{5}$	highway interchange	$v_{14}$	Slovakia border cross
$v_{6}$	Radom	$v_{15}$	Lublin
$v_{7}$	Łódź	$v_{16}$	Belarus border cross
$v_{8}$	Warszawa	$v_{17}$	Białystok
$v_{9}$	Gdańsk	$v_{18}$	Szczecin

**Table 2:** The length ${\text{l}}_{\text{k}}$ of each edge ${\text{e}}_{\text{k}}$ in the road network.

Edge ${\text{e}}_{\text{k}}$	Length ${\text{l}}_{\text{k}}$	Edge ${\text{e}}_{\text{k}}$	Length ${\text{l}}_{\text{k}}$
*e*_1_	1.44	*e*_13_	2.95
*e*_2_	1.87	*e*_14_	1.40
*e*_3_	1.69	*e*_15_	0.96
*e*_4_	1.71	*e*_16_	0.56
*e*_5_	0.69	*e*_17_	0.75
*e*_6_	1.72	*e*_18_	1.16
*e*_7_	2.25	*e*_19_	1.75
*e*_8_	2.11	*e*_20_	2.45
*e*_9_	0.93	*e*_21_	2.89
*e*_10_	2.86	*e*_22_	2.46
*e*_11_	1.84	*e*_23_	2.82
*e*_12_	1.18	*e*_24_	2.70

To evaluate the edge percolation centrality as a generic centrality measure for contagion spread, a complex contagion model is simulated over *t* = 100 time steps, with the contagion initiating from a specific peripheral vertex. Let $S_{v},I_{v}$, and $R_{v}$ denote the population of susceptible, infected, and removed individuals, respectively, at vertex v∈V and time *t*>0 such that the contagion spreads according to the diffusive Susceptible-Infected-Removed (SIR) model developed in [18]


$$S_{v}^{\prime }(t)=-\tau_{v}S_{v}(t)I_{v}(t)$$

$$I_{v}^{\prime }(t)=\tau_{v}S_{v}(t)I_{v}(t)-\eta_{v}I_{v}(t)+\sum_{e|e∼v}\alpha_{e}^{v}I_{e}(v,t)-\left(\sum_{e|e∼v}\lambda_{e}^{v}\right)I_{v}(t)$$

$$R_{v}^{\prime }(t)=\eta_{v}I_{v}(t)$$

with the boundary condition at the vertices


$$D_{v}\partial_{x}\underset{―}{I_{e}}+K_{v}\underset{―}{I_{e}}=\Lambda_{v}\underset{―}{I_{v}}$$

and the diffusion equation on edge *e*


$$\partial_{t}I_{e}=d_{e}\partial_{x}^{2}I_{e}$$

where $D_{v}$ and $K_{v}$ are matrices that encode the coupling and transfer rates at vertex *v*, and *I*_*e*_ is the line density of traveling infected population along edge *e*. The parameters used in this model are described in Table 3. Details on the appropriate parameter ranges and on the construction of the matrices used in the boundary condition can be found in [18].

**Table 3:** The description of the parameters from the diffusive SIR model.

Parameter	Description
$\tau_{v}$	Transmission rate at vertex *v*
$\eta_{v}$	Removal rate at vertex *v*
*d*_*e*_	Diffusion coefficient for edge *e*
$\lambda_{e}^{v}$	Rate of individuals leaving vertex *v* to travel to edge *e*
$\alpha_{e}^{v}$	Rate of individuals leaving edge *e* and traveling to incident vertex *v*

For example, consider a specific simulation in which peripheral vertex $v_{18}$ is initially infected. (An aside, $v_{18}$ depicts the largest city near the first documented case of COVID in Poland in 2020.) Specifically, each populated vertex, $\left\{v_{i}\in V\;|\;i\neq 18\right\}$, starts with $S_{v}^{0}=0.25$ and $I_{v}^{0}=0$ while $S_{v_{18}}^{0}=0.249999$ and $I_{v_{18}}^{0}=0.000001$. The border crossing vertices have $S_{v}^{0}=I_{v}^{0}=0$ and remain zero for the duration of the simulation. The parameters are set to the following values:


$$\tau_{v}=0.45,\;\;\eta_{v}=0.13$$

while


$$\lambda_{e}^{v}=\frac{0.1}{\deg(v)},\;\alpha_{e}^{v}=0.125,\;d_{e}=1.5\;\forall v\in V,\;\forall e\in E$$

and


$$v_{e_{m},e_{n}}^{v}=0.1\;\forall e_{m},e_{n}\in E\;\forall v\in V$$

In addition, the time discretization is set to Δt=0.02 while the space discretization to Δx=0.55 for each e∈E.

**Fig 9:**
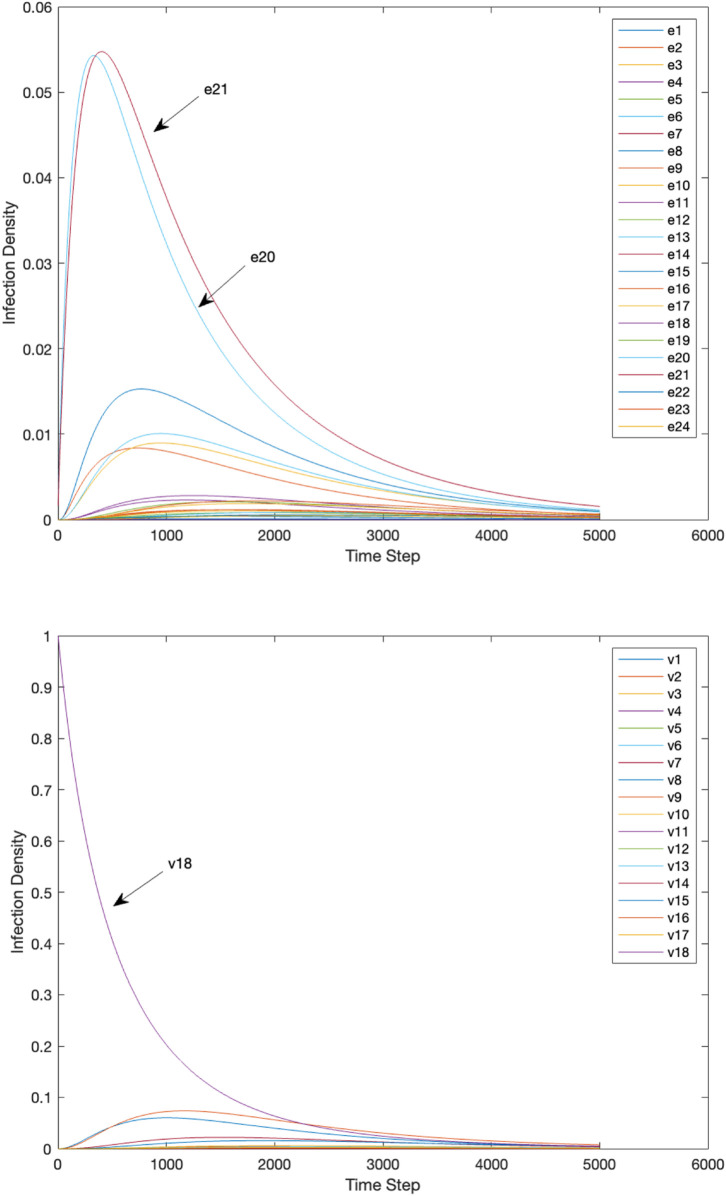
The infection density for each edge (top) and vertex (bottom) over time in the road network. The *x*-axis shows discrete time steps used in the numerical simulation with time step size △t=0.02.

At each time step *t*, the contagion diffuses from vertex to vertex through the edges. As the contagion spreads, the percolation states of the edges are updated based upon the infection density ($0\leq I_{e}^{t}\leq 1$) at time step *t*. Fig 9 illustrates the spread of the contagion through the edges and vertices of the network over time, beginning at *t* = 1 with the initial infection of a single vertex and culminating at *t* = 100. Vertex $v_{18}$ and edges *e*_20_ and *e*_21_ have the highest infection densities; this is not surprising as the contagion started at $v_{18}$, which is connected only by *e*_20_ and *e*_21_.

**Fig 10:**
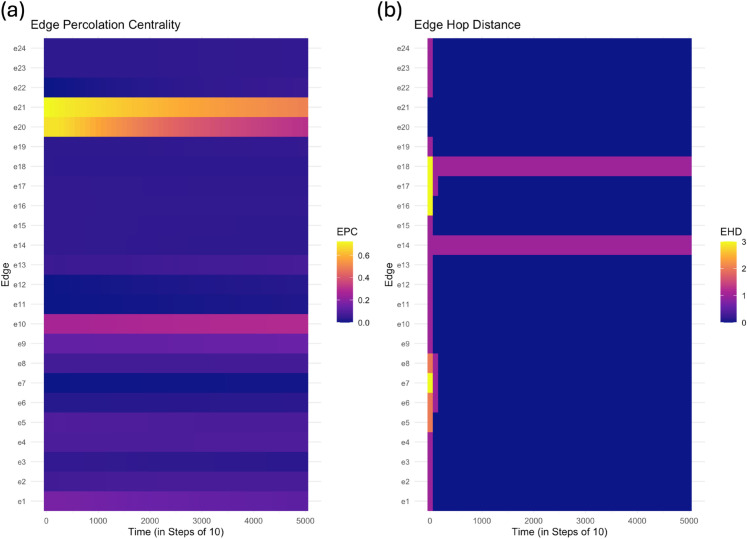
The EPC (a) and EHD (b) of each edge in the road network over time (in steps of 10) with the contagion initiated on vertex ${\text{v}}_{18}$. Brighter colors indicate larger values. The *x*-axis shows discrete time steps used in the numerical simulation with time step size △t=0.02.

Fig 10(a) illustrates the EPC of each edge in the road network over time (in steps of 10) while Table 4 displays the mean EPC of each edge. Overall, edge *e*_21_ exhibits the highest EPC, with elevated values appearing early in the contagion spread and gradually tapering off. Edge *e*_20_ also shows elevated EPC values, though lower than *e*_21_. Edge *e*_10_ maintains a moderate but persistent EPC throughout the contagion spread, while most other edges (e.g., $e_{3},e_{7},e_{11}$) exhibit only low EPC values.

**Table 4:** The average EPC values (rounded to four significant figures) of the edges in the road network.

Edge	Mean EPC	Edge	Mean EPC
*e*_1_	0.0757	*e*_13_	0.0794
*e*_2_	0.0712	*e*_14_	0.0375
*e*_3_	0.0452	*e*_15_	0.0441
*e*_4_	0.1054	*e*_16_	0.0644
*e*_5_	0.1053	*e*_17_	0.0531
*e*_6_	0.0728	*e*_18_	0.0391
*e*_7_	0.0210	*e*_19_	0.0833
*e*_8_	0.0765	*e*_20_	0.1581
*e*_9_	0.1855	*e*_21_	0.2907
*e*_10_	0.2630	*e*_22_	0.0643
*e*_11_	0.0520	*e*_23_	0.0909
*e*_12_	0.0789	*e*_24_	0.0823

To contrast edge percolation centrality, edge hop distance (EHD) is calculated for each edge over time in the road network. Fig 10(b) shows the EHD of each edge, where lower values indicate proximity to infected edges. Edges *e*_20_ and *e*_21_ exhibit consistently low EHD throughout the simulation given their direct involvement in the initial spread of contagion. As the contagion advances, several edges (e.g., *e*_8_ and *e*_17_) become more exposed over time such that EHD transitions from higher to lower values. Conversely, a few edges (i.e., *e*_14_ and *e*_18_) maintain high EHD values for most of the simulation due to their geographic distance from the infected region.

Together, the EPC and EHD results highlight complementary aspects of edge significance during contagion spread. Edges with high EPC and low EHD values (e.g., *e*_20_ and *e*_21_) play a central role in facilitating transmission and are located near the origin of the outbreak. In contrast, edges with low EPC and consistently high EHD values (e.g., *e*_14_ and *e*_18_) remain largely uninvolved in the spread due to their geographic distance from infected regions. Other edges (e.g., *e*_1_ and *e*_10_) display moderate EPC with decreasing EHD values over time, indicating a delayed but eventual exposure to the contagion. Collectively, these results suggest that EPC captures an edge’s sustained influence in transmission dynamics, while EHD reflects its evolving proximity to active transmission zones.

To explore whether removing or “blocking off” high EPC edges can effectively reduce the spread of an epidemic, the influence of each edge removal is evaluated by measuring the total change in the area under the infection curves (AUC) of each vertex across the road network (see Table 5). Specifically, the AUC is computed by numerically integrating each vertex’s infection density over time using the trapezoidal rule via the trapz function in Matlab [19], applied to infection curves sampled every 10 time steps and scaled to population-level values.

**Table 5:** The total change in the area under the infection curve (Δ AUC) across all vertices for each edge removed in the road network.

Edge	Δ AUC	Edge	Δ AUC
*e*_1_	29.6019	*e*_13_	2.3562
*e*_2_	1.4817	*e*_14_	1.2526
*e*_3_	0.0685	*e*_15_	0.1099
*e*_4_	–1.2850	*e*_16_	0.0486
*e*_5_	–0.5398	*e*_17_	0.0888
*e*_6_	–0.2979	*e*_18_	0.5219
*e*_7_	0.2384	*e*_19_	0.5766
*e*_8_	0.3245	*e*_20_	13.4281
*e*_9_	–0.6173	*e*_21_	–14.8271
*e*_10_	–0.4837	*e*_22_	0.2857
*e*_11_	0.6623	*e*_23_	0.3143
*e*_12_	14.5113	*e*_24_	0.2580

Notably, removing *e*_21_ (the edge with the highest mean EPC) results in the most significant decrease in AUC (–14.8271), suggesting its removal effectively reduces the overall infection spread. In contrast, removing *e*_20_, despite being a critical edge connecting the infection origin ($v_{18}$) to other parts of the network, leads to a positive change in AUC (13.4281). Interestingly, the six edges with negative Δ AUC values form a path ($e_{21}-e_{10}-e_{9}-e_{6}-e_{5}-e_{4}$) connecting $v_{18}$ to $v_{7}$, a vertex located in the geographical center of the network.

Although *e*_20_ and *e*_21_ are both adjacent to the initial infection source, they play distinct roles in the contagion’s progression. Removing *e*_21_, a major pathway to the geographical center of the network, significantly delays and diminishes the spread, leading to a sharp reduction in AUC. In contrast, removing *e*_20_ forces the contagion to reroute through *e*_21_ and other efficient paths. While this delays the peak, it also causes the contagion to remain longer, resulting in more sustained transmission and a higher cumulative infection, thus increasing the AUC.

## Discussion

In this work, a new edge centrality measure, the edge percolation centrality (EPC), is proposed to analyze the influence of edges in a network undergoing a percolation process. Developed as an analogue to the vertex percolation centrality proposed by [10], EPC measures the extent that individual (partially or fully) percolated edges influence the percolation process in the network at any given time. When all edges are fully percolated (or partially percolated to the same extent), EPC reduces to the edge betweenness centrality. In the best-case scenario, EPC can be computed in $O(|V{|}^{4})$ time, which is computationally slower than the edge betweenness centrality (as noted in [20], edge betweenness can be calculated in a time that scales as O(|V||E|)). However when some edges are (partially or fully) percolated, then EPC is effective in identifying the relative influence of edges on further percolation; in such conditions, edge betweenness (and other static edge centrality measures) remains ineffective.

To demonstrate how EPC can be utilized as a generic measure applicable in the context of any contagion spread, two contagions (one simple and one complex) are simulated over real-world networks. The first simulation involves a network of 39 vertices obtained from a contact network study in Alberta, Canada; here, the contagion spreads in a simple manner where edges become percolated with a transmission probability of *p* = 0.20. The second simulation involves a network of 18 vertices modeling a road network in Poland; here, the contagion spreads according to the diffusive SIR model developed by [18]. In both simulations, EPC identifies influential edges within the percolation process. Yet the second simulation demonstrates the utility of EPC as an intervention tool to mitigate contagion spread. Specifically, removing the edge with the highest mean EPC reduces the overall contagion spread across the network. By emphasizing the topological position of an edge and its changing percolation state over time, EPC serves as a valuable tool for understanding and managing percolation processes in networks.

Possible directions for future work include reducing the computational complexity of EPC to enhance its applicability to large networks, extending EPC to weighted networks, and integrating EPC into optimization frameworks for real-time intervention strategies. In addition, prior studies have shown that community structure can significantly affect spreading dynamics and that incorporating community-level information can improve the identification of influential vertices [21–23]. Consequently, investigating whether similar approaches can be applied to EPC represents another direction for future work.
